# Cyclin-dependent kinases as mediators of aberrant transcription in prostate cancer

**DOI:** 10.1016/j.tranon.2025.102378

**Published:** 2025-03-30

**Authors:** Razia Rahman, Luke A. Selth

**Affiliations:** a Flinders University, College of Medicine and Public Health, Flinders Health and Medical Research Institute, Adelaide, South Australia; b Flinders University, Freemasons Centre for Male Health and Wellbeing, Adelaide, South Australia; c Faculty of Health and Medical Sciences, The University of Adelaide, Adelaide, Australia

**Keywords:** Prostate cancer, Cyclin-dependent kinase, Androgen receptor, Targeted therapies, Transcription

## Abstract

Transcriptional control of gene expression is fundamental to all cellular processes. Conversely, transcriptional dysregulation is a hallmark of cancer. While this hallmark is a key driver of all malignancy-related process, it also represents a vulnerability that can be exploited therapeutically. Prostate cancer is a prime example of this phenomenon: it is characterised by aberrant transcription and treated with drugs that influence transcriptional pathways. Indeed, the primary oncogenic driver and therapeutic target of prostate cancer, the androgen receptor (AR), is a transcription factor. Moreover, a plethora of other transcriptional regulators, including transcriptional cyclin-dependent kinases (CDK7, CDK8 and CDK9), MYC and Bromodomain-containing protein 4 (BRD4), play prominent roles in disease progression. In this review, we focus on the roles of transcriptional CDKs in prostate cancer growth, metastasis and therapy resistance and discuss their interplay with AR, MYC and BRD4. Additionally, we explore recent advances in the therapeutic targeting of transcriptional CDKs and propose how these strategies could be effectively harnessed for the treatment of prostate cancer.

## Highlights

• Dysregulated transcription is a hallmark of prostate cancer.
• The androgen receptor (AR), a transcription factor that is the primary therapeutic target in advanced prostate cancer, is a key driver of dysregulated transcription in prostate cancer. However, aberrant activity of multiple other transcriptional regulators, including transcriptional cyclin-dependent kinases (CDKs), MYC and BRD4, is also critical for prostate cancer growth, metastasis and therapy resistance.
• The interplay between AR, transcriptional CDKs, MYC and BRD4 is intricate and orchestrates many malignancy-associated processes.
• Targeting transcriptional CDKs with small-molecule inhibitors has emerged as a major therapeutic strategy in cancer. This strategy is the subject of extensive preclinical research and many clinical trials in prostate cancer.
• Transcriptional CDK inhibitors may have the greatest impact on patient care when applied in combination with other anti-cancer treatments, including hormonal therapies and immunotherapies.

## Graphical abstract

**Figure:**
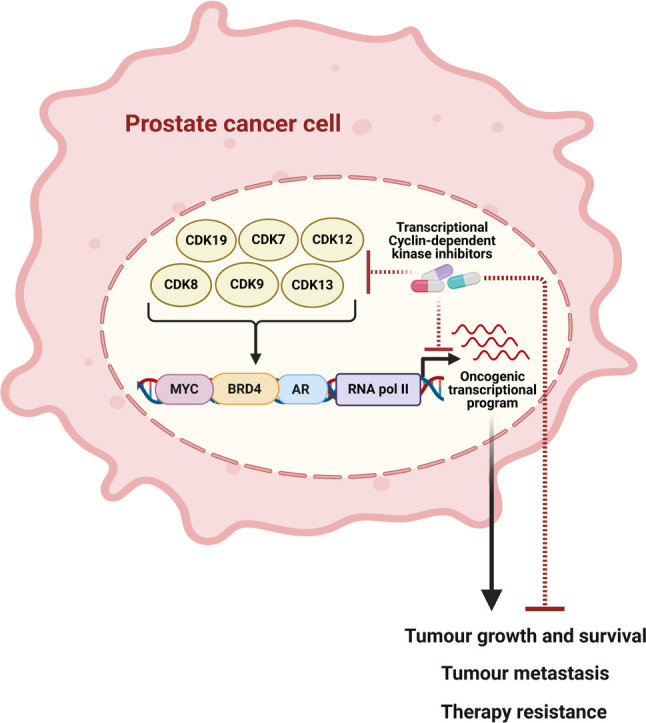

## Introduction

Dysregulation of transcriptional control is a hallmark of malignancy. Aberrant gene expression, the gain of hyperactive regulatory elements - such as super-enhancers – and increased overall transcription collectively enable phenotypic adaptations that influence tumour growth, survival, metabolism and evasion of the immune system [[1], [2], [3]]. The dependency of cancer cells on dysregulated transcriptional programs has been termed “transcriptional addiction” [1]. Emerging evidence suggests that transcriptionally-addicted cancer cells are highly sensitive to factors that disrupt transcription, providing opportunities for therapy [1,[3], [4]].

Prostate cancer is the second most common malignancy in men worldwide and a leading cause of cancer-related death [5]. A hallmark of prostate cancer is its dependence on the androgen signalling axis and the androgen receptor (AR) [6]. Therefore, the key treatment strategy for advanced prostate cancer is to inhibit AR activity. AR-targeted therapies include: i) drugs that reduce circulating and intra-tumoural androgen levels, such as androgen deprivation therapy with luteinizing hormone-releasing hormone agonists/antagonists or the androgen biosynthesis inhibitor abiraterone acetate; and ii) AR antagonists (anti-androgens) that block the binding of androgens to AR, such as bicalutamide, enzalutamide, darolutamide and apalutamide [[7], [8], [9], [10], [11]]. While AR-targeted therapies provide a survival benefit for most men, prostate tumours inevitably develop mechanisms of resistance and progress to a lethal disease state termed castration-resistant prostate cancer (CRPC) [[12], [13], [14]].

Most castration-resistant tumours exhibit highly active AR signalling [[12], [13], [14]]. Persistence of AR signalling despite ongoing targeted therapy is a result of alterations to this pathway, including *AR* gene amplification/over-expression, *AR* mutations, the emergence of constitutively active AR variants, altered expression of AR co-regulators and increased intratumoral androgen synthesis [15]. Beyond AR, altered expression and activity of other transcriptional and epigenetic regulators, including MYC, Bromodomain-containing protein 4 (BRD4) and Enhancer of zeste homolog 2 (EZH2), is commonly observed during disease onset and progression [[16], [17], [18]]. In a smaller subset of tumours, AR activity is lost and enhanced cellular plasticity confers phenotypes – such as neuroendocrine and stem-like states – that promote metastasis, therapy resistance, immune evasion and other oncogenic features [19,20].

Another class of transcriptional regulators with emerging roles in prostate cancer growth and therapy resistance are the transcriptional cyclin-dependent kinases (t-CDKs). In normal cells, t-CDKs orchestrate the transcription cycle via a series of sequential biochemical reactions that control RNA synthesis and processing. However, it is increasingly apparent that t-CDKs underlie important oncogenic pathways in most tumour types [[21], [22], [23], [24]]. In prostate cancer, t-CDKs appear to play a particularly prominent role because they also facilitate the activity of malignancy-associated transcription factors such as AR, MYC and BRD4 [1,[3], [4], [25],^26^]. Below, we describe the relevance of t-CDKs to prostate cancer in more detail and illuminate possibilities for targeting this class of factors to improve treatment.

## Regulation of transcription by cyclin-dependent kinases

**Fig. 1:**
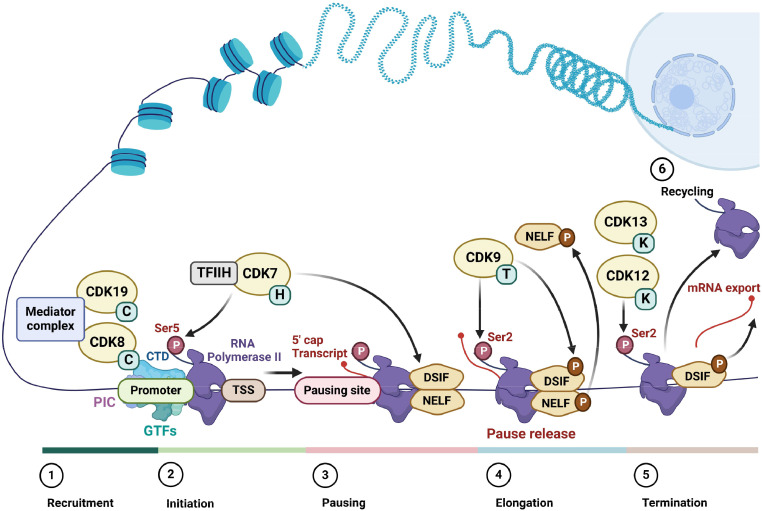
**The role of transcriptional CDKs in regulating RNA pol II-mediated transcription.** The RNA pol II transcription cycle can be divided into four main sequential steps – initiation, pausing, elongation and termination. Initiation begins with recruitment of RNA pol II and the general transcription factors (GTFs) and the assembly of the pre-initiation complex (PIC). The CDK8/Mediator complex binds to PIC, which anchors it to gene-specific transcription start sites (TSS). CDK7 facilitates promoter escape by phosphorylating RNA Pol II carboxy-terminal domain (CTD) at serine 5 (Ser5), promoting the recruitment of pre-mRNA 5′capping enzymes. Subsequently, RNA pol II enters a promoter-proximal pausing state mediated by its association with DRB sensitivity-inducing factor (DSIF) and negative elongation factor (NELF). To proceed to a productive elongation state, CDK9 is recruited to acetylated chromatin, which in turn phosphorylates DSIF, NELF, and the RNA Pol II CTD at serine 2 (Ser2), which collectively enables RNA pol II pause release. CDK12/CDK13 maintain the progression of transcription across the gene body by further phosphorylating RNA pol II Ser2. CDK12 also regulates transcriptional termination by the recruitment of cleavage and polyadenylation factors. Following termination of transcription, RNA pol II is recycled for another cycle of transcription. Created with BioRender.com.

CDKs regulate critical cellular events including the cell cycle, gene transcription and metabolism. The so-called transcriptional CDKs - CDK7, CDK8, CDK9, CDK10, CDK11, CDK12, CDK13 and CDK19 along with their activating cyclin partners - play a vital role in regulating gene expression [21,23]. Transcription is a multi-step sequential process involving pre-initiation, initiation, elongation and termination stages, all of which are regulated by RNA polymerase II (RNA pol II), the general transcription factors (GTFs), CDKs and other co-activator proteins [27] (Fig. 1). The carboxy-terminal domain (CTD) of the catalytic RPB1 subunit, the largest subunit of RNA pol II, is composed of tandem heptapeptide repeats (52 repeats in humans) of the consensus sequence Tyrosine-Serine-Proline-Threonine-Serine-Proline-Serine (YSPTSPS) that are essential for its function [28]. The number of repeats in the CTD partially determines the processing efficiency of the different pre-mRNA substrates, while dynamic phosphorylation of amino acids within the repeats directs distinct RNA pol II-driven transcriptional outputs [29].

### Regulation of transcription initiation by CDK8/CDK19

The first intermediate step preceding transcription initiation is the formation of the pre-initiation complex (PIC) at the gene promoter, which comprises RNA pol II, GTFs including TFIIA, TFIIB, TFIID/TATA-binding protein (TBP), TFIIE, TFIIF and TFIIH, and the multiprotein Mediator coactivator complex [30]. The Mediator complex interacts with DNA-bound transcription factors and RNA Pol II, facilitating the assembly of the PIC at transcription start sites (TSS) (Fig. 1). CDK8 (or its paralogue CDK19) plays a central role in regulating PIC formation. CDK8 is the catalytic subunit of the CDK8 kinase module (CKM), which is a dissociable sub-component of Mediator that also includes cyclin C, MED12, and MED13. The CKM phosphorylates the RNA pol II CTD to influence formation and stability of the PIC as well as pausing of RNA pol II [[31], [32], [33], [34]].

### Regulation of transcription initiation and pausing by CDK7

CDK7 associates with cyclin H and the accessory protein MAT1 to form the heterotrimeric CDK-activating kinase (CAK) complex, which is a part of the core TFIIH transcription initiation complex [35,36]. CDK7/CAK has dual functions in the transcription process – stimulating initiation of transcription via the core TFIIH complex and mediating transcriptional pausing [[37], [38], [39]]. After the formation of the PIC and following unwinding of DNA by helicases, CDK7 facilitates the removal of the CDK8-containing mediator complex by phosphorylating RNA Pol II CTD at its Serine 5 (Ser5) residue [40,41] (Fig. 1). This leads to activation of mRNA synthesis by RNA Pol II and its release from the PIC [[27], [29]].

Subsequently, following transcription of 20–60 nucleotides downstream of the TSS, CDK7 promotes recruitment and phosphorylation of DRB sensitivity-inducing factor (DSIF) and negative elongation factor (NELF), both of which are essential for promoter-proximal pausing of RNA pol II [35] (Fig. 1). Pausing serves multiple purposes: it increases gene accessibility; it enhances accurate deciphering of gene-specific transcription regulatory signals; and it serves as a checkpoint for the quality control of nascent pre-mRNA via 5′ end-capping, which stabilises the transcript from degradation [42,43].

### Regulation of transcription elongation by CDK9

To proceed to productive transcriptional elongation, the activity of the CDK9-cyclin T complex, P-TEFb, is crucial for the release of paused RNA pol II [42]. P-TEFb is recruited to paused RNA pol II where it phosphorylates both DSIF and NELF, prompting NELF ejection from chromatin and converting DSIF into a positive elongation factor [[44], [45], [46], [47]] (Fig. 1). P-TEFb then phosphorylates RNA Pol II at Serine 2 (Ser2) of the CTD, which faciliates its release from promoter-proximal pausing [48]. Ser2 phosphorylation also leads to recruitment of additional CTD-binding proteins that facilitate transcription by assisting with chromatin remodeling and RNA processing [[49], [50], [51], [52], [53]].

### Regulation of transcription elongation and termination by CDK12 and CDK13

CDK12 [54] and CDK13 [55] both form a complex with cyclin K and can also phosphorylate the RNA pol II CTD at Ser2 (Fig. 1). Although both of these kinases have the same substrate as CDK9, they primarily function in later transcriptional elongation processes and do not appear to have a significant role in early transcriptional events [56]. More specifically, CDK12/CDK13 sustain Ser2 phosphorylation levels towards the middle and 3′-end of genes to enable productive transcript synthesis [57,58]. Additionally, CDK12 regulates the processing of nascent mRNA by promoting the use of distal 3′ transcription termination sites and suppressing intronic polyadenylation, resulting in the production of full-length gene transcripts [59]. CDK12 also regulates 3′ transcription termination, since Ser2 phosphorylation influences the actiivty of polyadenylation factors that are required for this end phase of transcription [60]. CDK13′s role in transcription is less well understood but is thought to be gene-selective and distinct from CDK12, despite their conserved domain structure and shared interaction partners [55,61]. In addition to phosphorylating RNA pol II CTD, CDK12 and CDK13 phosphorylate various pre-mRNA processing and RNA splicing factors that play key roles in both transcription and other processes; however, the precise consequences of these post-translational modifications remain to be fully elucidated [57,58,61].

### Other transcriptional CDKs - CDK10 and CDK11

The other transcriptional CDKs, CDK10 [62] and CDK11 [63], form complexes with cyclin M and cyclin L respectively. The functions and substrates of these kinases are not well characterised. CDK10 has been implicated as a tumour-suppressor in estrogen-driven cancers [62,64]. CDK11 has roles in alternative splicing, where it can influence splice site selection and associate with proteins involved in transcriptional initiation and elongation [[63], [65],^66^].

## Dysregulation of transcriptional cyclin-dependent kinases in prostate cancer

Genetic alterations and abnormal activity of t-CDKs are observed in most cancer types [67,68]. In prostate cancer, alterations to t-CDKs are very common [[68], [69]] (Table 1). CDK7 protein and mRNA levels are significantly higher in primary and metastatic prostate cancer compared to benign tissues and associated with disease recurrence after radical prostatectomy [70,71]. *CDK9* gene amplification occurs frequently in prostate cancer, particularly CRPC, and is associated with increased levels of this kinase [72]. Like CDK7, CDK9 expression is also predictive of more rapid disease recurrence following surgery [72].

**Table 1:** Altered mRNA and protein levels of transcriptional CDKs in prostate cancer.

CDK	Alterations	Disease stage	Reference
CDK7	Elevated mRNA expression	Primary	[70]
	Elevated protein expression	Primary or metastatic	[71]
CDK9	Elevated mRNA expression	CRPC	[72]
	Gene amplification	CRPC	
	Elevated protein expression	Primary	
CDK12	Gene mutation (biallelic loss)	All disease stages but more frequent in metastatic/CRPC	[73,74]
CDK8	Elevated mRNA expression	Metastatic	[75]
	Elevated protein expression	Metastatic or CRPC	[76]
	Gene amplification	CRPC	[77]
CDK19	Elevated mRNA expression	Primary or metastatic	[76]
	Elevated protein expression	Primary or metastatic or CRPC	[76]
	Gene amplification	CRPC	[77]

Biallelic loss-of-function mutations in the *CDK12* gene are present in primary prostate cancers and more often in metastatic CRPC [[74], [78], [79], [80]]. *CDK12* mutations are associated with rapid disease progression to metastatic and CRPC [[73], [81]]. CDK19 expression is also elevated in prostate cancer and correlates with shorter disease-free survival; interestingly, upregulation of this kinase appears to be specific to prostate cancer and not other tumour types [[76], [82]].

CDK8 is an androgen-repressed factor that remains unchanged in benign prostate tissue and primary prostate cancer. However, the frequency of *CDK8* gene amplification is highest in CRPC compared to other cancers and is associated with increased expression of CDK8 in metastatic disease [[75], [77],^83^].

## Oncogenic molecular activities of transcriptional cyclin-dependent kinases in prostate cancer

The oncogenic functions of CDK7 and CDK9 in prostate cancer are multifaceted. Inhibiting CDK7 induces potent growth inhibition and promotes cell cycle arrest, p53 activation and apoptosis [84]. CDK9 inhibition reduces aggressive prostate cancer cell growth by disrupting transcriptional elongation and AR-driven oncogenic programs and inhibits *in vivo* tumour growth [72,85]. Both CDK7 and CDK9 play a key role in enhancing transcription of oncogenes, many of which encode transcripts with short half-lives [[53], [84], [86], [87]]. One prominent example is the expression of genes encoding the BCL-2 family of anti-apoptotic proteins – BCL2, MCL1 and XIAP – which is enhanced by CDK9 activity and thereby enables cancer cell survival in a wide range of tumour types [[88], [89], [90], [91], [92]], including prostate cancer [[50], [86],^87^]. CDK7 and CDK9 also regulate the expression and activity of oncogenic transcription factors, including MYC (see below for more detail), AR (see next section for more detail), and nuclear factor-kappa B (NF-κB). Indeed, CDK9/P-TEFb acts as an essential coactivator of NF-κB to enable transcription of anti-apoptotic and pro-inflammatory genes [93].

CDK12 can act as a tumour suppressor by maintaining genomic stability via regulating DNA damage response (DDR) genes [94]. Accordingly, *CDK12* mutations are associated with genomic instability in many cancer types, including prostate cancer [[58], [81], [95]]. However, the functions of CDK12 in prostate cancer are complex and pleiotropic. For example, a recent study identified CDK12 to be critical for prostate cancer cell survival and its inhibition suppressed AR signalling [96]. Moreover, inhibiting CDK12 can be a strategy to induce DNA damage, resulting in reduced cell proliferation and induction of G2/M arrest and apoptosis [97]. Beyond prostate cancer, it has been reported that CDK12 can activate the non-canonical NF-κB pathway by enhancing expression of NFκB-inducing kinase in osteosarcoma cells [98]. Given the critical role of NF-κB in mediating androgen-independent phenotypes in prostate cancer [99], it is tempting to speculate that CDK12 may also play a role in prostate cancer progression and therapy resistance by stimulating this factor, although this concept requires further investigation.

Increased levels of CDK8 and CDK19 are associated with a higher migratory potential in prostate cancer cell lines [76,77]. A recent study showed that CDK8/CDK19 inhibition reversed the castration-resistant phenotype of CRPC tumours in vivo by modifying the oncogenic transcriptional responses to castration. Additionally, prolonged CDK8/CDK19 inhibition downregulated the MYC pathway leading to tumour regression [100].

## Regulation of ar by transcriptional cyclin-dependent kinases

**Fig. 2:**
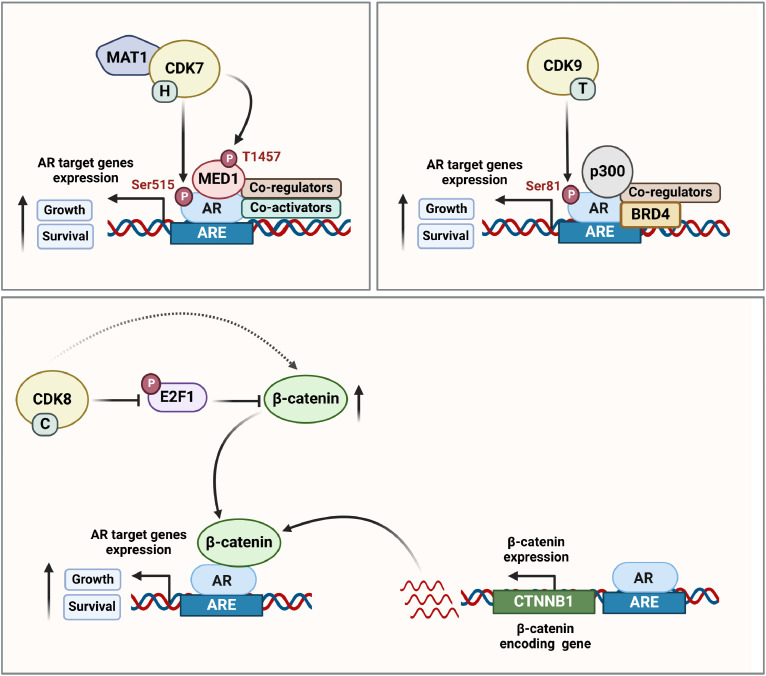
**Regulation of AR by transcriptional CDKs.** (Top left) CDK7 directly phosphorylates AR at serine 515 (Ser515) or indirectly phosphorylates AR by phosphorylating MED1 (Mediator Complex Subunit 1) at Threonine 1457 (Thr1457), which promotes AR's interaction with co-regulator proteins and increases its transcriptional activity. (Top right) CDK9 directly phosphorylates AR at serine 81 (Ser81), which facilitates its association with chromatin and regulates AR's transcriptional activity. (Bottom) CDK8 increases β-catenin expression involved in WNT signalling by inhibiting E2F1 and elevated CDK8 regulates AR activity. Additionally, AR regulates the expression of the β-catenin-encoding gene (*CTNNB1*) resulting in a positive feedback loop between AR and β-catenin. Collectively, t-CDKs promote the association of AR at the androgen response elements (ARE) and enhance the recruitment of AR co-activators, thereby driving AR transcriptional activity. Created with BioRender.com.

Apart from regulating transcription of key cancer-associated genes in prostate tumours, t-CDKs also play critical roles in AR expression and activity. Indeed, AR is a direct substrate of multiple t-CDKs and its phosphorylation influences protein stability, nuclear localisation and transcriptional output. CDK7 phosphorylates AR at Serine 515 (Ser515) in the N-terminal domain, which promotes recruitment of AR to chromatin and increases its transcriptional activity, both of which may be explained by phosphorylation-dependent changes to AR's interaction with co-regulator proteins [101,102] (Fig. 2). AR phosphorylation at Ser515 also regulates AR turnover by the ubiquitin-proteasome system at gene promoters, which is important for its transcriptional activity [101].

CDK9 primarily phosphorylates AR at its Serine 81 (Ser81) residue, which facilitates its nuclear retention and association with chromatin and also regulates promoter selectivity, effects that collectively enhance AR's transcriptional activity [103,104] (Fig. 2). As evidence of the importance of this post-translational modification, mutation of Ser81 reduced AR-regulated gene transcription and suppressed cancer cell growth [104]. Phosphorylation of AR at Ser81 (AR pSer81) also enhances the recruitment of key co-activators – for example, the histone acetyltransferase p300 and BRD4 – which assists in releasing P-TEFb to initiate a positive feedback loop that maintains transcription of AR target genes [105,106]. The influence of CDK9 on AR pSer81 has been shown in both castration-sensitive and castration-resistant cell lines models of prostate cancer [[103], [104],^107^]. In CRPC mice xenografts, AR pSer81 was decreased following castration but then increased again with the onset of castration resistance and restoration of AR activity [108]. Ser81 is in the AR N-terminal domain, which is also present in constitutively active AR variants that lack the ligand-binding domain. Accordingly, phosphorylation of this residue was found to influence transcriptional activity of the AR-V7 variant, resulting in activation of pro-metastatic transcriptional programs [109].

Apart from influencing AR activity directly, t-CDKs also indirectly influence AR activity. For example, CDK7 phosphorylates Threonine 1457 (T1457) of the Mediator Complex Subunit 1 (MED1), which is upregulated in prostate cancer cells [110] (Fig. 2). MED1 acts as an AR coactivator that promotes its engagement with cancer-associated enhancers and super-enhancers, and this activity is increased by MED1 T1457 phosphorylation [[71], [111]]. Loss of MED1 phosphorylation due to CDK7 knockdown leads to a reduction in MED1 recruitment to AR-bound enhancers and super-enhancers, resulting in dampening of target gene expression and reduced growth of CRPC xenografts [112]. CDK8 also regulates AR indirectly by increasing the expression of β-catenin, a key effector molecule of the WNT signalling pathway that is often dysregulated in CRPC and linked to therapy resistance. More specifically, CDK8 phosphorylates and inhibits E2F1, a factor that promotes β-catenin protein degradation [113]. Increased levels of β-catenin due to elevated CDK8 can enhance AR activity, since β-catenin acts as a transcriptional co-activator [114]. Interestingly, the β-catenin-encoding gene (*CTNNB1*) can be induced by ligand-bound AR [115], creating a positive feedback loop between these two factors [83,116] (Fig. 2).

## Interplay between oncogenic transcription factors and transcriptional cyclin-dependent kinases in prostate cancer

### MYC

Transcriptional CDKs regulate the activity of numerous transcription factors that are highly relevant to prostate cancer growth and progression. Of particular interest is CDK9′s role as a regulator of MYC, a well-studied transcription factor that promotes the growth and progression of many cancer types [[117], [118], [119], [120]]. CDK9 plays critical roles in both the upstream and downstream regulation of MYC [[121], [122], [123]]. CDK9 directly regulates *MYC* gene expression, particularly in cancer contexts [[124], [125], [126]]. The resulting high levels of MYC protein increase its binding to DNA; this in turn facilitates CDK9 recruitment to enhancers and promoters and thereby enhances transcription of the MYC-regulated gene program [[88], [122],^127^,^128^]. Interplay between these two factors is further reinforced by direct phosphorylation of MYC serine 62 by CDK9, which enhances MYC protein stability [129]. The relationship between CKD9 and MYC has clinical ramifications: the high dependency of MYC-driven tumours on CDK9 render them more sensitive to CDK9 inhibitors [130], a concept that is being harnessed in clinical trials of these drugs whereby patients are selected for treatment based on *MYC* amplification status (see Table 2 and below for more detail).

**Table 2:** Active clinical trials of transcriptional CDK inhibitors.

Target	Inhibitor	Cancer type	Trial phase	Status	Trial identifier
CDK7	Q901	Advanced solid tumours - advanced or metastatic ovarian cancer - castration-resistant prostate cancer - hormone receptor-positive, HER2-negative breast cancer - endometrial cancer - colorectal cancer - small cell lung cancer - pancreatic cancer that has progressed following standard-of-care therapy	1/2	Recruiting	NCT05394103

CDK9	CYC065 (Fadraciclib)	Advanced solid tumours and lymphoma with *MCL1* or *MYC* amplification - endometrial cancer - ovarian cancer - biliary tract cancer - hepatocellular carcinoma - breast cancer: hormone receptor-positive, HER2-negative, HER2-refractory metastatic, triple-negative - B-cell lymphoma - T-cell lymphoma - metastatic colorectal cancer	1/2	Recruiting	NCT04983810

	SLS009	Relapsed/refractory haematological malignancies - acute myeloid leukemia - chronic lymphocytic leukemia, small lymphocytic lymphoma and lymphoma	1/2	Recruiting	NCT04588922

KB-0742	Relapsed/refractory solid tumours with *MYC* copy number gain - epithelial ovarian cancer - triple-negative breast cancer - non-small cell lung cancer - small cell lung cancer - non-Hodgkin lymphoma - B-cell lymphoma with *MYC* translocation - Ewing's sarcoma - alveolar rhabdomyosarcoma - NUT midline carcinoma - chordoma	1	Recruiting	NCT04718675

CDK8	RVU120	Relapsed/refractory acute myeloid leukemia	1	Recruiting	NCT06268574

		Acute myeloid leukemia or high-risk myelodysplastic syndrome	1	Active (not recruiting)	NCT04021368

Intermediate or high-risk, primary or secondary myelofibrosis	1	Not yet recruiting	NCT06397313

**Fig. 3:**
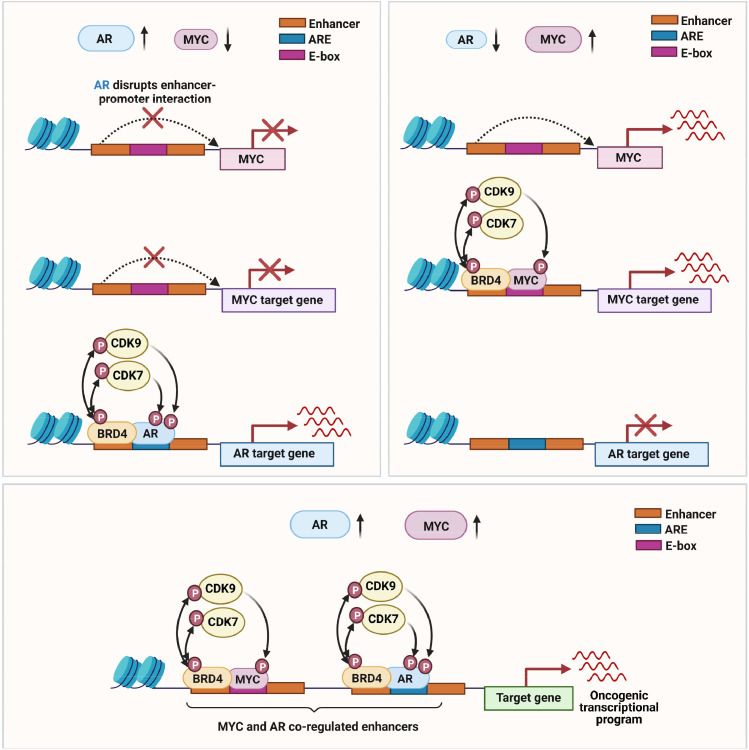
**A model of the interplay of transcriptional CDKs, AR, MYC and BRD4.** (Top left) AR overexpression leads to repression of MYC transcription by disrupting the MYC enhancer-promoter interaction and binding to enhancer regions required for AR target gene expression. (Top right) MYC overexpression leads to repression of AR transcription and increased MYC expression and its target genes. Furthermore, the interaction between t-CDKs and BRD4 facilitates AR or MYC-driven oncogenic transcriptional programs. (Bottom) Binding of MYC indirectly facilitates the association of AR with enhancers, promoting an oncogenic transcriptional program. Created with BioRender.com.

MYC is a key driver of both AR-dependent and AR-independent CRPC but its relationship with AR is complex [131]. In general, this relationship is considered to be antagonistic. AR can repress MYC transcription by suppressing the *MYC* enhancer as well as by sequestering co-factors required for *MYC* gene transcription; therefore, inhibition of AR activity leads to increased *MYC* expression [132,133] (Fig. 3). Conversely, elevated levels of MYC diminishes the AR transcriptional program in pre-clinical models and CRPC tumours, at least in part by disrupting transcriptional pause release at AR target genes [126] (Fig. 3). However, the relationship between these factors is not always antagonistic; indeed, in certain situations, they can act in a concerted manner to promote tumour growth. Evidence for oncogenic cooperation between MYC and AR includes the observation that MYC can stimulate AR's association with enhancer elements (including super-enhancers) that regulate a cancer-associated transcriptional program [[126], [134],^133^] (Fig. 3) as well as the more recent finding that AR may directly promote transcription of the *MYC* gene [135]. In short, there remains much to understand about the context-dependent relationship between MYC and AR (Fig. 3); given the central role of these factors in prostate cancer, further research in this area is certainly warranted.

The input of t-CDKs has profound implications for AR-MYC interplay. For example, CDK7 is an important regulator of both factors, at least in part because it phosphorylates MED1 to enhance AR- and MYC-regulated transcription in prostate cancer [136]. Accordingly, CDK7 inhibition blocks the activity of both AR and MYC, which is associated with reduced CRPC xenograft growth [84,112]. By contrast, CDK8/CDK19 enhance AR activity and suppress MYC expression; therefore, inhibition of these kinases leads to dampening of AR signalling concomitant with MYC upregulation, resulting in aberrant G1/S transition, activation of a DNA damage response and significant anti-tumour activity in CRPC xenografts [137].

### BRD4

BRD4 is a transcriptional and epigenetic regulator that is frequently overexpressed in prostate cancer, particularly CRPC, and associated with aggressive clinical features [138,139]. BRD4 contains bromodomains that recognise acetylated lysine residues on histones and other target proteins, leading to its association with hyper-acetylated chromatin [140]. BRD4 is a key regulator of CDK9: it directly phosphorylates CDK9 at either Threonine 29 to repress its activity or Threonine 186 to activate its activity. Therefore, depending on the relative levels of phosphorylated Threonine 29/186, BRD4 alternatively represses or activates the kinase activity of CDK9 [[141], [142], [143], [144]]. The interplay between these factors is bidirectional, since CDK9 also phosphorylates BRD4 at residues in its N-terminal and C-terminal regions [145,146]. A key consequence of blocking the BRD4-CDK9 interaction is downregulation of target genes, including MYC [[72], [115]]; indeed, the regulation of MYC expression and function is tightly connected with BRD4 at the transcriptional and post-transcriptional levels [17]. Similarly to CDK9, CDK7 also has a bidirectional relationship with BRD4 whereby both are substrates for each other, creating a regulatory loop that is important for gene transcription in cancer [141] (Fig. 3).

**Fig. 4:**
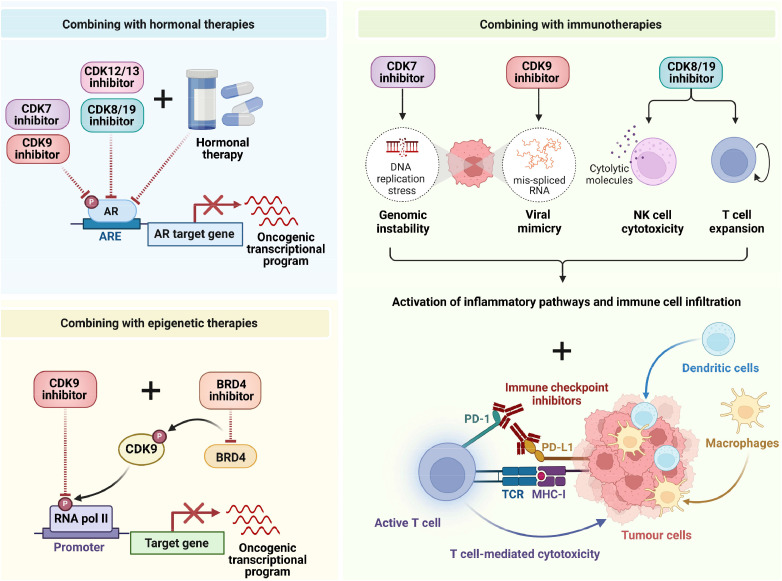
**Emerging combination therapies incorporating t-CDK inhibitors.** Potential combination treatment strategies involving combined inhibition with current standard-of-care hormonal therapies (top left), epigenetic therapies (bottom left) and immunotherapies (right). BRD4, Bromodomain-containing protein 4; PD-1, Programmed cell death protein 1; PD-L1, Programmed death-ligand 1; TCR, T cell receptor; MHC-I; Major histocompatibility complex class I. Created with BioRender.com.

AR activity in prostate cancer is also intimately linked to BRD4. AR-driven prostate cancer models are preferentially sensitive to BRD4 inhibition, since BRD4 and AR co-localise at target loci, including super-enhancers, to drive AR-mediated gene transcription [[16], [147]]. CDK9-directed phosphorylation of AR Ser81 enhances BRD4 recruitment, which in turn enhances CDK9 activity, creating a positive feedback loop that maintains AR transcriptional activity [106,148]. Another observation reflecting the intimate association between BRD4, CDK9 and AR in prostate cancer is that cells that have acquired resistance to BRD4 inhibition exhibit adaptive responses to AR and CDK9 [149]. More specifically, these BRD4 inhibitor-resistant cells exhibit increased CDK9 activity and thereby elevated levels of AR pSer81, a phenomenon that renders them more sensitive to CDK9 inhibition and AR blockade [149]. Collectively, these findings support the concept of co-targeting t-CDKs and BRD4, which is explored in more detail in Fig. 4 and below.

## Regulation of the tumour microenvironment by transcriptional cyclin-dependent kinases

Prostate cancer cells are surrounded by a complex ecosystem that comprises other non-cancer cells – such as fibroblasts, endothelial cells, immune cells and adipocytes – and an extracellular matrix, collectively termed the tumour microenvironment (TME) [150,151]. The TME plays an integral role in prostate cancer pathogenesis, metastasis and resistance to therapy [[151], [152]]. Since t-CDKs are ubiquitously expressed, it is important to consider their functions in non-cancer cells and how their targeting could influence the TME. The effects of inhibiting t-CDKs on T cell function has been investigated in several cancer types, including prostate cancer (see discussion below on combining t-CDK inhibitors with immunotherapies for more detail). Beyond T cells, several studies have characterised the impact of t-CDK inhibition on other components of the TME. Inhibition of CDK8/CDK19 was shown to influence gene expression in host stroma of mice harbouring CRPC xenografts and these transcriptional changes were tentatively linked to tumour suppression [100]. Moreover, CDK18/CDK19 inhibition suppressed the tumour-promoting activities of fibroblasts [153] and decreased intra-tumoural blood supply in an AR-driven patient-derived xenograft model, the latter finding suggesting that these kinases enhance tumour angiogenesis [100]. Interestingly, multiple other t-CDKs have been implicated in tumour angiogenesis: inhibition of CDK7/CDK9 suppresses vascular endothelial growth factor (VEGF)-induced angiogenic effects on endothelial cells [[154], [155]], while CDK12-mediated activation of VEGF and PI3K/AKT/mTOR signalling has been implicated in angiogenesis in prostate and gastric cancer, respectively [156,157].

Although these studies are instructive, our analysis of the literature revealed that the field's understanding of t-CDK function in the TME remains limited. In prostate cancer, this knowledge gap is important to consider in the context of AR function, since AR is also expressed, and has important cancer-associated functions, in many non-tumour TME cells i.e. adaptive immune cells (B and T cells), innate immune cells (macrophages and monocytes), fibroblasts and smooth muscle cells [158]. In short, elucidating t-CDK function in the TME, including its interplay with AR, will be critical to advance the field's understanding of prostate cancer pathobiology as well as the development of t-CDK-based therapeutic approaches.

## Transcriptional cyclin-dependent kinases as therapeutic targets in prostate cancer

In the last two decades, several small molecule inhibitors of t-CDKs have been developed as potential therapeutics and evaluated in multiple cancer types, several of which have progressed to clinical trials [[25], [159],^160^]. Table 2 lists the t-CDK inhibitors that are currently in clinical trials. The early inhibitors were somewhat promiscuous, targeting multiple CDKs: flavopiridol (alvocidib), which inhibits CDK1, 2, 4, 6, 7, and 9; roscovitine (seliciclib), an inhibitor of CDK1, 2, 5, 7 and 9; and dinaciclib, an inhibitor of CDK1, 2, 5 and 9. Although these drugs exhibited activity against haematological malignancies in clinical trials, severe toxicities and limited therapeutic windows precluded their clinical deployment [[26], [159],^161^]. A poor understanding of the molecular mechanisms of action of the pan-CDK inhibitors, at least partly resulting from their lack of specificity, has caused additional issues that have hindered their use [[26], [159]]. To overcome these issues, more selective and potent inhibitors have been developed in the recent years, as described below.

### CDK7 inhibitors

The first highly-selective CDK7 inhibitor, BS-181, suppressed growth of breast cancer mice xenografts *in vivo* [162]. BS-181 was later structurally optimised to yield an orally bioavailable CDK7 inhibitor, ICEC0942 (samuraciclib). Preclinical studies showed that ICEC0942 was an effective anti-cancer agent across multiple tumour types [163,164]. In prostate cancer, samuraciclib inhibited cell and tumour growth by targeting proliferative pathways and AR signalling and inducing apoptosis [84]. Samuraciclib has completed phase I clinical studies for advanced solid malignancies, including CRPC and triple-negative breast cancer (TNBC) (NCT03363893) and in combination with a hormonal therapy (fulvestrant) in HR+/HER2- breast cancer patients post failure of CDK4/6 inhibitor therapy [[112], [165]]. It demonstrated an acceptable pharmacokinetic and safety profile with initial evidence of efficacy and antitumor activity, particularly in CRPC [165].

Another CDK7 inhibitor, THZ1, has been validated in several preclinical *in vitro* and *in vivo* tumour models [[166], [167], [168], [169]]. In prostate cancer, THZ1 caused tumour regression of AR-driven CRPC mouse xenograft models by suppressing AR-dependent oncogenic transcription programs [112]. Optimisation of THZ1 led to a more metabolically stable analogue, SY-1365 [170]. A phase I clinical trial of SY-1365 revealed that clinical activity was achieved only with higher or more frequent doses, which would be overly burdensome for patients [171]. As such, clinical development of SY-1365 was discontinued and focus shifted to another orally bioavailable analogue, SY-5609, which has potent antitumour activity [172,173]. A phase I clinical trial of SY-5609 was recently completed in advanced solid tumours (NCT04247126), however, no results have been reported yet. Another analogue of THZ1, YKL-5–124, is highly selective for CDK7 [174] and a recent study revealed its activity in rapidly proliferating CRPC cells [70].

Another potent and orally bioavailable covalent CDK7-selective inhibitor, XL102 (formerly known as AUR102), has shown promise as an anti-cancer therapy. Preclinical studies of XL102 in models of breast cancer, prostate cancer, and lymphoma demonstrated that it induced cell death and resulted in tumour regression [175]. XL102 is currently being evaluated in a clinical trial for advanced or metastatic solid cancers (NCT04726332) and preliminary data showed drug tolerance at the doses evaluated. The efficacy of XL102 will be further investigated as a single-agent dose-escalation study (solid tumour) and in combination with abiraterone/prednisone in metastatic CRPC [176].

### CDK9 inhibitors

Development of CDK9 inhibitors has been an active area of oncology research with several selective inhibitors being developed in recent years. BAY1143572 (atuveciclib) was the first selective CDK9 inhibitor to enter clinical trials. BAY1143572 showed marked anti-tumor activity in acute myeloid leukemia (AML) preclinical models [177] and adult T-cell leukaemia [178]. It has been evaluated in two cohorts of patients with AML (NCT02345382) and advanced solid tumours (NCT01938638). A modified structure of atuveciclib, termed VIP152, has greater potency in preclinical models and was well tolerated in mice [179,180]. VIP152 is being tested in a phase I clinical trial for advanced solid cancers and aggressive non-Hodgkin's lymphoma (NHL) (NCT02635672); the preliminary findings revealed a favourable safety profile and therapeutic activity [181].

AZD4573 (AstraZeneca) is another highly potent CDK9 inhibitor with >25-fold selectivity for CDK9 over other CDKs; it has demonstrated broad anti-tumour activity in preclinical studies and in a clinical trial of relapsed haematological malignancies (NCT03263637) [182,183]. Preclinical studies with fadraciclib, another selective CDK9 inhibitor, showed that cell lines expressing high MCL1 or a high MCL1:BCL2L1 ratio are the most sensitive to this agent [[184], [185], [186]]; however, the potential of these factors as predictive biomarkers requires validation in prospective clinical trials. Fadraciclib is currently being tested in a phase I clinical study for patients with advanced solid tumours and lymphoma who have progressed following standard-of-care therapies (NCT04983810). CDKI-73 is another novel orally bioavailable CDK9 inhibitor that is ∼6–80 fold more selective for CDK9 than other CDKs (CDK1, 2 or 7) [187]. Several preclinical studies have demonstrated its potential utility in models of leukemia, ovarian cancer, colorectal cancer, melanoma and prostate cancer [[72], [188], [189], [190], [191], [192]].

KB-0742 is another orally bioavailable selective CDK9 inhibitor that has shown promising activity against prostate cancer. It caused decreased phosphorylation of AR Ser81, resulting in suppression of AR activity that was linked to reduced growth of CRPC xenografts [85]. Moreover, KB-0742 reduced tumour burden in MLL-rearranged AML *in vivo* models [85]. These findings encouraged the ongoing phase I/II trial of KB-0742 in patients with advanced solid tumours (NCT04718675).

### CDK8/CDK19 inhibitors

A number of small-molecule CDK8/CDK19 inhibitors have been developed and their anti-cancer effects demonstrated in preclinical studies of multiple tumour types [193]. Cortistatin A, a natural marine product, was the first selective CDK8 inhibitor reported and was found to reduce growth of AML cell lines [194]. Cmpd3 and senexin A and an optimised derivative, senexin B, have been shown to suppress growth of ER-positive breast cancer mice xenografts as well as sensitising these tumours to fulvestrant [195]. Preclinical studies with T-474 and T-418, two structurally distinct CDK8/CDK19 inhibitors, demonstrated induction of prostate cancer cell death and significant anti-tumour activity in CRPC xenografts [137]. Recently, a study reported reduced migration and invasion of different prostate cancer cell lines in response to two other CDK8/CDK9 inhibitors, CCT251545 and G02788177.93–1 [[76], [77],^196^]. Subsequently, another study revealed a novel CDK8 inhibitor, E966–0530–45,418, that potently inhibited migration of prostate cancer cells and demonstrated anti-metastatic properties in CRPC xenografts [75]*.*

The only selective CDK8 inhibitors to progress to clinical trials are RVU120 (previously named SEL120) [197] and BCD-115 (previously named senexin B) [198]. The anti-cancer activity of these inhibitors was initially demonstrated in leukaemia models. Subsequently, BCD-115 was the first CDK8 inhibitor to enter clinical trials for the treatment of locally advanced or advanced metastatic breast cancer (NCT03065010). Later, RVU120 was trialled in acute myeloid leukemia (NCT04021368). Although it resulted in the stabilization of metastases in patients, it exhibited metabolic instability and adverse effects in patients [198]. However, a phase I/II study of RVU120 as single agent for relapsed or refractory metastatic or advanced solid tumours was subsequently announced (NCT05052255).

### CDK12/CDK13 inhibitors

Although interest in CDK12/CDK13 inhibition has increased in the recent years, there is a lack of selective small molecule chemical probes and drug-like compounds targeting these kinases. The covalent CDK7 inhibitor THZ1, which also has activity against CDK12/CDK13 at higher doses, was used to develop a covalent CDK12/CDK13-selective inhibitor, THZ531, and its optimised derivative, BSJ-01–175 [199,200]. THZ531 reduced global transcription in Jurkat T-ALL cells. Low doses of THZ531 downregulated DDR-related gene expression, while higher doses of THZ531 suppressed super-enhancer-regulated transcription factors and induced apoptosis [200]. In a recent study, THZ531 blocked cellular proliferation, induced apoptosis and DNA damage and decreased expression of DDR-related genes in prostate cancer cell lines [97]. Another study showed that THZ531 preferentially downregulated oncogenic transcripts that are sensitive to CDK12 inhibition and/or associated with super-enhancers, thereby downregulating survival processes in prostate cancer cells [96]. However, to our knowledge, none of these CDK12/CDK13 inhibitors have been evaluated in clinical trials yet.

## Emerging combination therapies incorporating inhibitors of transcriptional cyclin-dependent kinases

Although many of the t-CDK inhibitors discussed above have shown favourable antitumour activity as single agents, the exploration of combination treatment strategies with existing or standard treatments will facilitate their optimal clinical deployment (Fig. 4). Combination strategies may be particularly efficacious in transcriptionally-addicted cancers, since these malignancies exhibit high heterogeneity that requires inhibition of multiple targets to effectively restrict disease progression [3]. Combination therapies also facilitate use of lower drug doses, minimising the potential of treatment-related toxicities.

### Combining t-CDK inhibitors with hormone therapies

A potentially promising combination therapeutic strategy is combining t-CDK inhibitors with therapies that target sex hormone signalling axes (i.e. AR-targeted therapies in prostate cancer and equivalent therapies targeting the estrogen receptor in breast cancer) (Fig. 4). In the context of prostate cancer, given the interplay of t-CDKs and AR in regulating the transcriptional output of genes involved in cell proliferation and survival, combining t-CDKs inhibitors with hormone therapies is a rational approach that has been shown to augment anti-cancer activity in preclinical studies [84,97,96,163]. Inhibitors of t-CDKs may also be useful in delaying or preventing the inevitable onset of castration-resistant states by inhibiting alternative pathways and transcription factors that drive resistance.

Preclinical studies of the CDK7 inhibitor CT7001 in breast cancer models provided evidence that combining CT7001 with tamoxifen (hormonal therapy) resulted in better antitumour activity compared to CT7001 alone [163]. Preclinical evidence also supports combining t-CDK inhibitors with antiandrogens in prostate cancer. Indeed, synergy between the CDK8/CDK19 inhibitor G02788177.93–1 and the AR antagonist bicalutamide has been reported: the combination dramatically reduced LNCaP cell viability in a time-dependent manner while G02788177.93–1 or bicalutamide treatment alone had only modest antiproliferative effects that stabilised over time [196]. Similarly, combining the CDK12/CDK13 inhibitor THZ531 with AR inhibition enhanced anti-proliferative and pro-apoptotic activities in androgen-sensitive prostate cancer cells [97]. Inhibition of CDK12 has also been shown to synergise with multiple antiandrogens (enzalutamide, apalutamide and bicalutamide); mechanistically, CDK12 inhibition caused AR down-regulation and augmented epigenetic suppression of AR target genes [96]. Similarly, the anti-tumour effects of samuraciclib in CRPC xenografts were significantly augmented when combined with enzalutamide [84]. CDK9 inhibitors are also logical candidates for use in combination with antiandrogens, since CDK9 inhibition was shown to suppress AR signalling in several studies [[53], [72], [103], [104], [201]].

### Combining t-CDK inhibitors with immunotherapies

There has been a growing interest in the use of t-CDK inhibitors as immunomodulatory and immunosensitisation agents [[3], [202],^203^] (Fig. 4). Mechanisms by which t-CDK inhibitors promote anti-tumour immunity are beginning to be elucidated. By promoting DNA replication stress and genome instability, the CDK7 inhibitor YKL-5–124 triggered a pro-inflammatory response that facilitated recruitment of effector CD4+ T cells, cytotoxic CD8+ T cells and dendritic cells to the TME and augmented the efficacy of an anti-PD-1 immune checkpoint inhibitor [204]. A more recent study found that CDK7 inhibition with samuraciclib increased expression of the immune checkpoint protein PD-L1 and increased infiltration of M1 macrophages and CD8+ cytotoxic T lymphocytes into TNBC tumours, which improved the efficacy of a PD-L1 inhibitor [164]. A clinical trial that combines another CDK7 inhibitor, SY-5609, with the anti-PD-L1 agent atezolizumab is underway in patients with *BRAF*-mutant colorectal cancer [205]. CDK9 inhibition can also drive inflammatory responses in a mechanism that involves accumulation of mis-spliced RNAs; these RNAs activate the dsRNA sensor PKR, leading to viral mimicry and NF-κB-mediated induction of cytokines [206]. Another mechanism by which CDK9 inhibition has been shown to increase anti-tumour immunity is by reactivating tumour suppressor genes that are important for cellular immune responses, which enhanced recruitment of immune cells to xenografts and sensitised a syngeneic ovarian cancer model to anti-PD-1 therapy [207]. CDK8/CDK19 inhibitors can also enhance anti-tumour immunity, with current evidence suggesting that they act primarily in a cancer cell-extrinsic manner. In natural killer (NK) cells, CDK8/CDK19 inhibition augments production of key cytolytic molecules to increase tumour kill and enhance response to anti-PD-1 immunotherapy [208]. A more recent study using CRISPR screening identified Mediator as a key regulator of human chimeric antigen receptor (CAR) T cell function and found that inhibition of CDK8/CDK19 enhanced expansion and activity of non-engineered T cells [209]. In prostate cancer, *CDK12*-mutant tumours exhibit higher T cell infiltration and expansion of T cell clones along with increased expression of chemokines and their receptors; accordingly, tumours with *CDK12* loss-of-function mutations showed a positive response to PD-1 inhibitors [210]. Another study found that high CDK12 levels was associated with increased expression of immune checkpoint factors PDCD1 and CTLA-4 in prostate cancer, supporting the concept of combining CDK12 inhibitors with immune therapy [211]. The potential of applying t-CDK inhibitors to sensitise tumours to immunotherapies is particularly attractive for prostate cancer since these tumours are immunologically “cold” and clinical trials of checkpoint inhibitors have been disappointing to date [212].

### Combining t-CDK inhibitors with epigenetic therapies

Reflecting the close association of CDK9 and BRD4 and their interplay in transcriptional regulation, co-targeting CDK9 and BRD4 has been found to elicit synergistic effects in multiple cancer types (Fig. 4). For example, the CDK9 inhibitor CDKI-73 and BET inhibitor IBET151 have shown synergistic effects in melanoma, MLL-rearranged leukemia and rhabdoid tumours [[188], [213],^214^]. Combination treatment with the CDK9 inhibitor LDC000067 and the BET inhibitor BI 894999 led to a strong reduction in global RNA pol II Ser2 phosphorylation levels and a marked induction of apoptosis *in vitro* and *in vivo*, and synergistically induced tumour regression compared to single agents [215]. Another study that combined CDK9 inhibitor MC180295 with decitabine, a specific DNA methyltransferase inhibitor, demonstrated synergistic effects in AML and colon cancer xenograft models [216]. In line with these findings, our group found that the CDK9 inhibitor, CDKI-73, inhibits BRD4 activity and reprogrammed cancer-associated super-enhancers in prostate cancer cells, and that its combination with the BRD4 inhibitor AZD5153 was synergistic in patient-derived organoids and *in vivo* models [72].

## Key points and future perspectives

Transcriptional CDKs have highly influential roles in the growth and development of prostate cancer, largely by regulating the activity of key oncogenic transcriptional regulators. As such, the potential of t-CDK inhibitors as a new treatment strategy for this disease is high. However, important biological and clinical questions remain to be addressed, which will facilitate the translation of such inhibitors to the clinic:

- Despite decades of research, we have an incomplete understanding of the functions of t-CDKs in normal cells and tissues; the gaps in knowledge are even greater when considering the oncogenic roles of these factors in cancer. Therefore, further basic research is required, particularly for understudied t-CDKs such as CDK10, CDK11, CDK12 and CDK13. Moreover, these studies should not be limited to the functions of t-CDKs in transcription but also focus on characterising their roles in other cellular processes and identifying additional protein substrates.
- It is increasingly apparent that t-CDKs influence the TME, not only by regulating transcription and splicing but also by affecting genome stability. However, the immunomodulatory activities of these kinases are poorly understood. This knowledge is crucial to harness t-CDK inhibition as a means to improve cancer immunotherapies, particularly in immunologically cold tumours such as prostate cancer. Cutting-edge omics technologies that enable comprehensive profiling of the TME, such as spatial and single cell transcriptomics and epigenomics, will facilitate progress in this area.
- Many t-CDK inhibitors have been developed and shown promise in laboratory studies. However, despite some of these progressing to clinical trials, there remains a large gap between basic scientific research and translation to novel therapeutics. This so-called “valley of death” is a major issue throughout oncology research but may be particularly problematic for t-CDK inhibitors, since these drugs target factors essential in most human tissues and hence require rigorous pharmacological evaluation. Strong relationships between academia and industry are required to increase the likelihood of advancing t-CDK inhibitors from the bench to the bedside.
- Identification of biomarkers that could predict response to t-CDK inhibition is essential for these therapies to impact on patient care. Some biomarkers have been identified and are already being evaluated in clinical trials: for example, *MYC* or *MCL1* amplification, both of which are known to enhance sensitivity to certain t-CDK inhibitors in preclinical models, have been or are being used to select patients for clinical trials (e.g., NCT04983810, NCT04718675). However, the clinical utility of these biomarkers is unproven and it is highly probable that other markers will improve patient stratification.
- How cancer cells acquire resistance to t-CDK inhibitors is poorly understood. Rigorous elucidation of resistance mechanisms – which will likely include epigenetic and genetic alterations, activation of alternative compensatory signalling pathways, effects on cell cycle dynamics and the TME – is required to optimise the clinical impact of these drugs. Preclinical research should be coupled with analysis of samples from clinical trials to address this question.
- Given the plethora of new drugs that have been approved to treat men with advanced prostate cancer in the past 2 decades, careful consideration of how t-CDK inhibitors would be deployed clinically is critical. It is highly likely that these agents would be applied in combination with AR-targeted therapies, necessitating further investigation of this combination strategy.

## Funding

This work was supported by grants from: 10.13039/501100001111Cancer Australia (2020_PdCCRS_2001432 and 2023_PCRS_0269); 10.13039/501100001102Cancer Council NSW (2020404); and The 10.13039/100009727Hospital Research Foundation (C-PJ-126–2019). LAS was supported by a 10.13039/100011040Principal Cancer Research Fellowship (PRF2919) awarded by Cancer Council's Beat Cancer project on behalf of its donors, the state Government through the Department of Health and the Australian Government through the Medical Research Future Fund. The research programs of RR are supported by the Australian Government Research Training Program Scholarship. The research programs of LAS are supported by the Flinders Foundation.

## CRediT authorship contribution statement

**Razia Rahman:** Writing – review & editing, Writing – original draft, Resources, Conceptualization. **Luke A. Selth:** Writing – review & editing, Writing – original draft, Supervision, Resources, Funding acquisition, Conceptualization.

## Declaration of competing interest

The authors declare that they have no known competing financial interests or personal relationships that could have appeared to influence the work reported in this paper.
